# Evolutions in Mentoring to Engineer Opportunity

**DOI:** 10.1093/geroni/igaf122.1240

**Published:** 2025-12-31

**Authors:** Sean Curran

**Affiliations:** University of Southern California Leonard Davis School of Gerontology, Los Angeles, California, United States

## Abstract

The Hiram J. Friedsam Award lecture will feature an address by the 2025 award recipient, Sean Curran, PhD, FGSA, Hiram J. Friedsam was the professor, co-founder, and director of the Center for Studies in Aging and dean of the School of Community Service at the University of Northern Texas. Dr. Friedsam was an outstanding teacher, researcher, colleague, and mentor to students, faculty, and administrators, as well as a past president of AGHE. The purpose of this award is to recognize those who emulate Dr. Friedsam’s excellence in mentorship.

